# Sympathetic Nervous Influences Are Negative Prognostic Factors in Stomach Cancer

**DOI:** 10.3390/life14030368

**Published:** 2024-03-11

**Authors:** Mihai Petrescu, Georgică Târtea, Ion Udriștoiu, Felicia Militaru, Alexandra-Roxana Petrescu, Ana-Maria Ciurea, Ana-Maria Petrescu, Cosmin Obleagă, Cristin Constantin Vere

**Affiliations:** 1 Department of Psychiatry, University of Medicine and Pharmacy of Craiova, 200349 Craiova, Romania; mihai.petrescu18@yahoo.com (M.P.); ion.udristoiu@umfcv.ro (I.U.); felicia.militaru@umfcv.ro (F.M.); 2 Department of Physiology, University of Medicine and Pharmacy of Craiova, 200349 Craiova, Romania; 3 Department of Pathology, University of Medicine and Pharmacy of Craiova, 200349 Craiova, Romania; alexandra.ciuciulete@yahoo.com; 4 Department of Oncology, University of Medicine and Pharmacy of Craiova, 200349 Craiova, Romania; amciurea14@gmail.com; 5 Department of Obstetrics and Gynecology, University of Medicine and Pharmacy of Craiova, 200349 Craiova, Romania; ana.petrescu048@gmail.com; 6 Department of Gastric Surgery, University of Medicine and Pharmacy of Craiova, 200349 Craiova, Romania; 7 Department of Gastroenterology, University of Medicine and Pharmacy of Craiova, 200349 Craiova, Romania; cristin.vere@umfcv.ro

**Keywords:** stomach cancer, sympathetic nervous influences, prognostic factors

## Abstract

(1) Background: The aim of our study was to assess the involvement of the sympathetic nervous system in the progression of patients with gastric carcinoma by analyzing the sympathetic neuronal fibers and beta 2 adrenoreceptors. (2) Methods: We performed a retrospective study in which we analyzed the clinical, biological, and histological data from a total of 104 patients diagnosed with stomach cancer. (3) Results: After analyzing the immunoreactivity of beta 2 adrenoreceptors, we observed increased values in patients with tumors larger than 5 cm in diameter (*p* = 0.0371), with a deeper degree of tumor invasion T3–4 (*p* = 0.0159), invasion in more than two lymph nodes (*p* = 0.0462), or a TNM stage III–IV. Regarding the survival analysis, better survival rates (65%) were observed for patients with a low value of beta 2 adrenoreceptors (B2A−), compared to B2A (+) patients, in which survival at 3 years of follow-up was only 43%. In addition, the analysis of intra-tumoral sympathetic fibers showed a better survival rate (83%) for patients with a low value of density compared to patients with increased density, in whom the survival rate was only 24%. (4) Conclusions: The findings of this study indicate that patients with stomach cancer have a more unfavorable prognosis when they have a higher density of sympathetic nerve fibers and an increased expression of beta 2 adrenergic receptors inside the tumor.

## 1. Introduction

Stomach cancer is the fifth most common type of cancer worldwide [1] and is mostly diagnosed between the sixth and the seventh decade of life [2]. The incidence is twice as high in men [1]. According to the latest data, in 2020, there were over one million new cases of gastric cancer worldwide, of which more than 75% were in Asia, and there were almost 800,000 deaths. It is estimated that by 2040, the number of new cases will increase to 1.77 million, with 1.27 million deaths [1]. According to the National Cancer Institute (USA), there is a 5-year relative survival rate for gastric cancer in 35.7% of the population (2013–2019) [2]. The highest mortality rates were observed in Eastern Asia for both males and females (21.1 vs. 8.8 per 100,000) [3]. Some of the factors identified that increase or might increase the risk of developing gastric cancer are poor diet (alcohol consumption, salt-preserved food, processed or grilled meat, and little or no fruit), obesity, smoking, Helicobacter pylori infection, and occupational chemical exposure (wood processing, food-machine operation, rubber, coal, or chromium production, and metal processing) [4]. Several environmental and genetic factors are involved, hence the interest in the evaluation of sympathetic nervous influences [5].

The enteric nervous system (ENS) is the most intricate component of the autonomic nervous system [6]. The enteric nervous system (ENS) is linked to the central nervous system via sympathetic and parasympathetic pathways, which are referred to as the gut–brain axis [7]. Neurotransmitters such as acetylcholine and norepinephrine facilitate connections between the parasympathetic nervous system (PNS) and sympathetic nervous system (SNS) divisions of the autonomic nervous system [6]. The role of the autonomic nervous system in the progression and development of gastric cancer is poorly understood, although multiple studies have demonstrated a strong correlation [8]. Batsakis et al. first demonstrated the existence of nerves in close proximity to human epithelial carcinomas, including gastric, head and neck, and prostate cancers, approximately 30 years ago [9,10]. The involvement of these nerves in the spread of cancer to other parts of the body through a process known as perineural invasion (PNI) has been documented [11]. PNI has been observed to be linked to a more aggressive tumor phenotype and unfavorable prognosis in various malignancies. A study on gastric cancer revealed that patients who tested positive for PNI had significantly shorter median survival compared to those who tested negative for PNI [12]. This study also demonstrated that PNI can be used as a valuable prognostic factor for curative gastric cancer. In contrast, Duraker’s study found that PNI did not offer any extra predictive information when compared to traditional parameters [13]. Recent research has demonstrated that the nervous system exerts both direct and indirect effects on the development and progression of gastric cancer. Gastric cancer cells infiltrate nerve fibers to stimulate an increase in neuronal cells and branching. In response, nerve fibers penetrate the tumor microenvironment to facilitate the advancement of gastric cancer [8]. Tumor cells release growth factors and exhibit elevated levels of catecholamines and multiple receptors [14,15,16]. The sympathetic nervous system (SNS) controls the activity of all human organ systems by releasing catecholamines from sympathetic nerve terminals in specific areas and by circulating catecholamines from the adrenal gland throughout the body [17]. These hormones have an impact on the functioning of immune cells and can also stimulate the growth, invasion, migration, and formation of blood vessels in tumors through specific biological pathways [18,19,20]. As a result, the activation of the sympathetic nervous system can control the expression of genes and the functioning of cells in the immediate environment of the tumor [17]. Noradrenaline (NE) plays a key role in the advancement of gastric cancer, primarily through its interaction with beta adrenergic receptors and the subsequent activation of various signal transduction pathways. Norepinephrine (NE) can trigger epithelial–mesenchymal transition (EMT), which in turn promotes the invasion and metastasis of gastric cancer [21,22,23,24]. The beta adrenergic pathway is responsible for mediating the sympathetic nervous system (SNS) [25]. Additionally, the beta adrenoreceptor system has been found to play a role in tumor progression [26,27]. Preclinical research has demonstrated that beta adrenergic antagonists effectively suppressed the spread of cancer to distant sites, whereas beta adrenergic agonists promoted the advancement of tumors, even in the absence of stress. These findings were reported in Refs. [28,29,30,31]. The data indicate that SNS signaling plays a role in the advancement and spread of malignant tumors. Tumor cells and nerves communicate with each other, resulting in the stimulation of new nerve cell growth. This leads to a higher concentration of nerve cells in tissues that are at risk of developing tumors [32,33,34,35]. The aim of this study was to assess the role of the sympathetic nervous system in the progression of patients with gastric carcinoma. This was undertaken by examining the sympathetic neuronal fibers and beta 2 adrenoreceptors and determining their relationship with clinical outcomes.

## 2. Materials and Methods

### 2.1. Patients

We conducted a retrospective study analyzing clinical, biological, and histological data from 104 patients diagnosed with stomach cancer. To avoid bias, each patient was consecutively enrolled in our study. These patients underwent surgery or upper digestive endoscopy and were treated at the Emergency Clinical Hospital of Craiova, affiliated with the University of Medicine and Pharmacy in Craiova, Romania, between January 2014 and November 2017. This study was conducted in accordance with the Declaration of Helsinki and other international regulations, and it received approval from the Ethics Committee of the University of Medicine and Pharmacy in Craiova (No. 225/20.12.2021). Prior to this study, the patients were provided with a comprehensive explanation of all stages involved. Written consent was obtained from the patients to ensure their voluntary participation. The patients were also informed about the confidential nature of the medical data and all study procedures were conducted in compliance with existing regulations, without interfering with normal diagnostic or treatment processes.

### 2.2. Histology

The histopathological examination was conducted following the surgical intervention or tumor biopsy obtained from the upper digestive endoscopy procedure. The biological material was added to a solution containing 10% formic aldehyde. The addition of calcium bicarbonate was used to neutralize the formic acid and achieve a neutral pH. The mixture was washed with water after the fixation procedure, followed by the embedding of the biological material in paraffin. Subsequently, dehydration occurred through the complete removal of water. This procedure was conducted by immersing the parts in ethyl alcohol solutions of varying concentrations, followed by an initial stage of embedding in paraffin. Subsequently, the clarification technique was used to remove alcohol from the tissue. The biological material was then placed in a series of paraffin baths and embedded in a paraffin block, resulting in its solidification. Ultimately, the paraffin blocks were divided into sections, the tissues were attached to the slides, hematoxylin–eosin staining was performed, and the histopathological diagnosis was independently conducted by two pathologists specialized in this domain to ensure accuracy.

### 2.3. Immunohistochemistry

The immunohistochemical analysis was conducted following the establishment of the histopathological diagnosis. The paraffin blocks containing the biopsy tissues were divided into sections using the HM350 rotary microtome (Thermo Fisher Scientific, Waltham, MA, USA). This microtome was equipped with a section transfer system in a cold-water bath and a Peltier cooling mode. The resulting slices were 3–4 μm thick. The slices were subsequently transferred to a water bath maintained at a temperature of 40 °C in order to achieve uniformity and elongation. The samples were retrieved from the water bath, which was heated to a temperature of 40 °C and placed onto slides coated with polylysine. The adhesion of the slices to the slides was enhanced by the presence of a positively charged amino acid residue. Following this protocol, the samples underwent a 24-h drying process in a thermostat set to 37 °C.

The following day, the slices were deparaffinized by sequentially immersing them in three xylene baths, each lasting approximately 15 min. Subsequently, the slices were rehydrated using alcohol solutions with progressively decreasing concentrations, until all traces of alcohol were eliminated by washing with distilled water.

The antigenic recovery process involved boiling for a total of 21 min, with each cycle lasting 3 min, in a microwave oven set at a power of 600 W. Boiling was carried out in a solution of sodium citrate with a pH of 6. Subsequently, the slices were subjected to a 30-min cooling process, followed by a 15-min washing procedure using both tap water and distilled water. To inhibit the endogenous peroxidase activity, the slices were exposed to a solution containing 1% hydrogen peroxide and distilled water for 30 min at room temperature. Subsequently, they were immersed in a solution of 3% skimmed milk powder in phosphate-buffered saline (PBS) for an additional 30 min. Finally, the slices were incubated with the primary antibodies at a temperature of 4 °C for 18 h.

The primary antibodies used in our study were anti beta 2 adrenoreceptors (B2A, clone NBP1-90227, dilution 1:100; Novus Biological, Abingdon, UK), tyrosine hydroxylase (TH, clone NB300-109, dilution 1:50; Novus Biological, Abingdon, UK), and anti Ki76 (clone MIB-1, dilution 1:50; Dako, Glostrup, Denmark).

To eliminate the surplus of primary antibodies, the following day, three consecutive PBS washes were conducted, followed by the application of secondary antibodies. The signal was enhanced for a duration of 30 min using a specialized system of peroxidase polymers that were absorbed by human immunoglobulins (Nikirei-Bioscience, Tokyo, Japan). The signal was identified using 3,3′-diaminobenzidine (DAB) from Dako, Glostrup, Denmark. Samples were then coated with DPX from Sigma-Aldrich in St. Louis, MO, USA, following staining with hematoxylin–eosin. All slices of tissue stained for each primary antibody were simultaneously processed to ensure protocol correlation. Control slices stained with either DAB or hematoxylin–eosin were included to obtain the distinct spectrum of colors. Negative controls were generated by excluding the primary antibodies.

### 2.4. Image Acquisition and Processing

To quantitatively assess the immunohistochemical expression of the target signal, while considering the histopathological aspect, we acquired light microscopy images using a Nikon Eclipse 90i motorized microscope (Apidrag, Bucharest, Romania). The microscope was outfitted with a Nuance FX multispectral camera and Nuance image analysis software (3.0.2) from Perkin Elmer, located in Hopkinton, MA, USA. First, an optical microscopy image was taken. Then, a composite image was created by overlaying color spectra for hematoxylin and DAB. As part of a subsequent procedure, distinct images were acquired for either the hematoxylin or DAB stains. The quantification of the unmixed DAB signal was performed by randomly selecting 10 images captured using a 20× objective. The integrated optical density (IOD) parameter was employed for the quantitative analysis of the color signal. To perform this analysis, we utilized the Image-Pro Plus AMS 7 image analysis software developed by Media Cybernetics, located in Bethesda, MD, USA. Using this software, we defined specific areas of interest, in which we analyzed the color signal and calculated the IOD. We manually excluded the stroma from the images we captured.

### 2.5. Clinicopathological Features

The patients included in our study were followed up for a period of 3 years, or until death. The clinicopathological features that were evaluated were age, gender, location of the tumor, histology, tumor size, tumor invasion, lymph node metastasis, and TNM stage.

### 2.6. Statistical Analysis

The data acquired using Image-Pro Plus AMS 7 image analysis software were exported to Microsoft Office Excel 2019 (Microsoft Corporation, Redmond, WA, USA) and analyzed using GraphPad Prism 9 software (San Diego, CA, USA). The results were reported using the mean and standard deviation. We employed Student’s *t*-test to compare the average values of the two groups. For the computation of the means of multiple groups, we employed the ANOVA variant analysis test. To establish the correlation between the various categories of data, the Pearson correlation test was employed. We employed the Log-rank test to examine the potential correlation between a variable and the duration of survival. Whenever the calculated *p*-value was less than 0.05, we deemed that a statistically significant difference existed between the means of the different groups being compared.

## 3. Results

### Clinicopathological Features

After analyzing the integrated optical density of beta 2 adrenoreceptors for the patients included in our study, we observed increased IOD values in patients with tumors larger than 5 cm in diameter (*p* = 0.0371), with a deeper degree of tumor invasion T3–4 (*p* = 0.0159), invasion in more than two lymph nodes (*p* = 0.0462), or a TNM stage III–IV (*p* = 0.0119). It is important to note that, in histological terms, there was no difference in the expression of beta 2 adrenoreceptors between adenocarcinoma and mixed carcinoma/signet ring cell carcinoma (*p* = 0.7107). The clinicopathological features assessed in our study are shown in Table 1.

**Figure 1:**
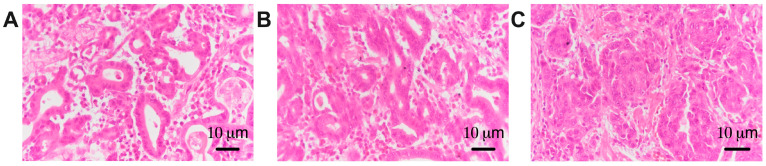
Gastric adenocarcinoma: well-differentiated—G1 (**A**), moderately differentiated—G2 (**B**), and poorly differentiated—G3 (**C**). Hematoxylin and eosin staining. Magnification: 20×.

The histological samples taken from patients with gastric cancer were analyzed independently by two pathologists with expertise in the field of gastric cancer and according to the degree of tumor differentiation: 23 patients were included in the group with well-differentiated tumors (G1), 49 patients were included in the group with moderately differentiated tumors (G2), and 32 patients were included in the group with poorly differentiated tumors (G3) (Figure 1).

**Figure 2:**
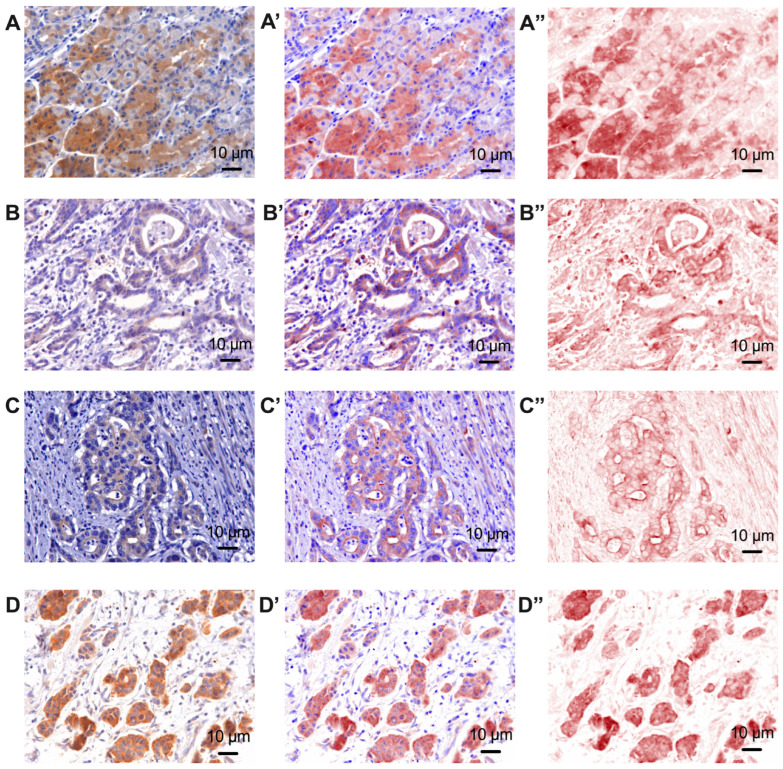
Representative pictures showing the immunoreactivity of beta 2 adrenergic receptors in normal peritumor gastric tissue (**A**) and in well-differentiated gastric tumor tissue—G1 (**B**), moderately differentiated tissue—G2 (**C**) and poorly differentiated tissue—G3 (**D**), 40×. (**A′**–**D′**) represent the mixed spectral compound. (**A″**–**D″**) represent the color channel only for beta 2 adrenoreceptors.

Depending on the degree of tumor differentiation, the immunoreactivity of beta 2 adrenergic receptors was analyzed (Figure 2). We observed a weaker signal for the expression of beta 2 adrenoreceptors in well-differentiated tumors compared to poorly differentiated tumors, where the color signal was much more intense.

**Figure 3:**
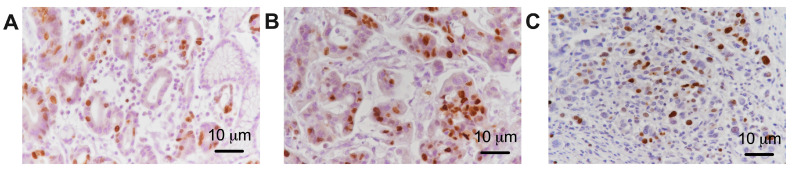
Representative pictures with the immunoreactivity of anti-Ki-67 in well-differentiated gastric tumor tissue—G1 (**A**), moderately differentiated tissue—G2 (**B**), and poorly differentiated tissue—G3 (**C**), 40×. The brown color represents cells in the process of proliferation without noting differences between the expression of the color signal depending on the degree of tumor differentiation.

**Figure 4:**
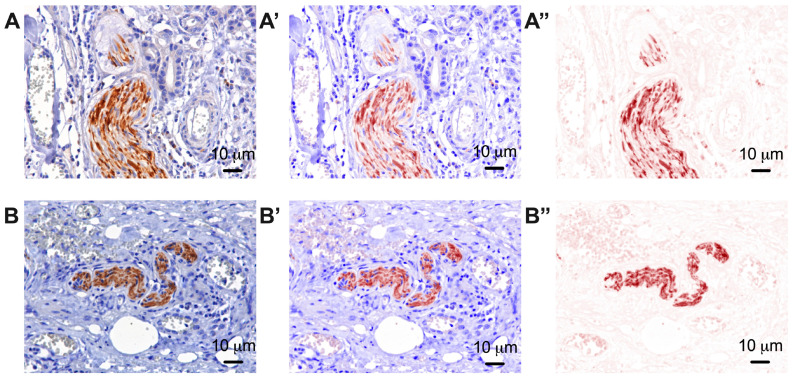
Representative pictures with the immunoreactivity of normal peritumor gastric tissue (**A**) and in gastric tumor tissue (**B**), 40×. (**A′**,**B′**) represent the mixed spectral compound. (**A″**,**B″**) represent the color channel only for tyrosine hydroxylase. The brown color represents the expression of this enzyme (tyrosine hydroxylase) in sympathetic nerve fibers. Moreover, a higher intensity of this enzyme is observed in intra-tumoral nerves (**B**) compared to extra-tumoral nerves (**A**).

In addition, considering the degree of tumor differentiation, the tumor proliferation index was evaluated by analyzing immunoreactivity for Ki 67 (Figure 3). For the analysis of the sympathetic nervous system in gastric cancer, we analyzed both the nerve fibers in the tumor tissues and the extra-tumoral nerve fibers by evaluating the percentage of tyrosine hydroxylase in each nerve fiber (Figure 4).

**Figure 5:**
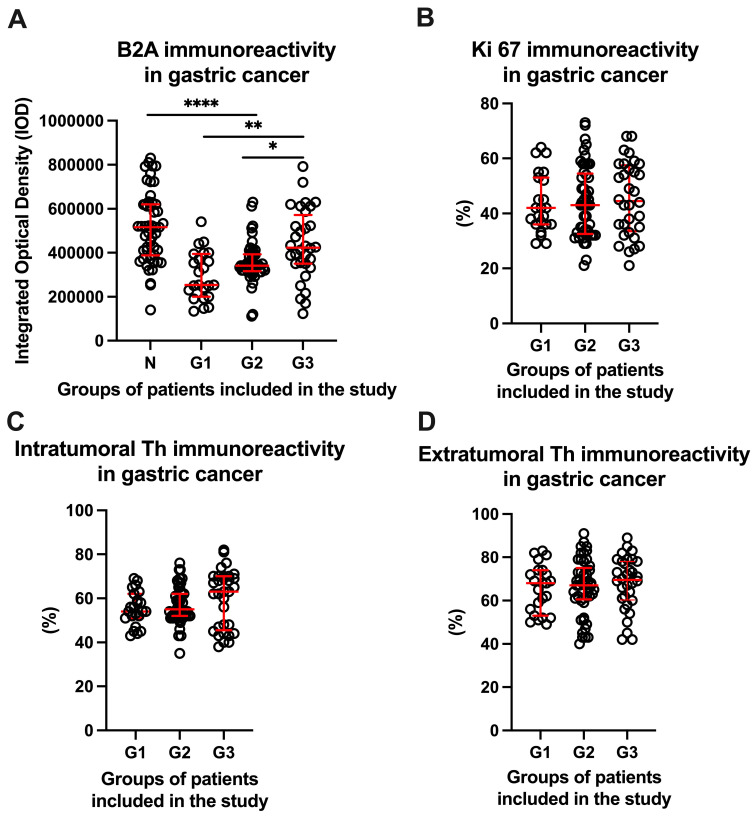
Immunoreactivity in gastric cancer for beta 2 adrenoreceptors—B2A (**A**), Ki 67 (**B**), and both intra-tumoral (**C**) and extra-tumoral (**D**) tyrosine hydroxylase. G1—well-differentiated gastric tumor tissue, G2—moderately differentiated tissue, and G3—poorly differentiated tissue. * represents statistically significant, ** represents highly significant and **** represents extremely significant.

Regarding the analysis of the immunoreactivity of beta 2 adrenergic receptors in gastric cancer, we observed an increase in them from well-differentiated tumors (297,704.0 ± 109,812.0) to moderately differentiated gastric tumors (357,031.0 ± 92,350.0) and poorly differentiated gastric tumors (438,204.0 ± 160,859.0). Moreover, compared to the gastric tissue, a decrease in the density of beta 2 adrenergic receptors was observed in patients with G1 and G2, respectively, which suggests that there is a statistically significant difference (*p* < 0.0001). This difference was not recorded between normal gastric tissue and poorly differentiated tumors (*p* = 0.0557). However, a statistically significant difference was observed both between poorly differentiated tumor tissue and well-differentiated tumors (*p* = 0.0013) and between poorly differentiated tumor tissue and moderately differentiated tumors (*p* = 0.0475). All these results are shown in Figure 5A. After analyzing the immunoreactivity of both intra-tumoral and extra-tumoral tyrosine hydroxylase, as well as the index of tumoral proliferation by immunoreactivity for Ki 67, no statistically significant differences were recorded depending on the degree of tumor differentiation (Figure 5B–D).

**Figure 6:**
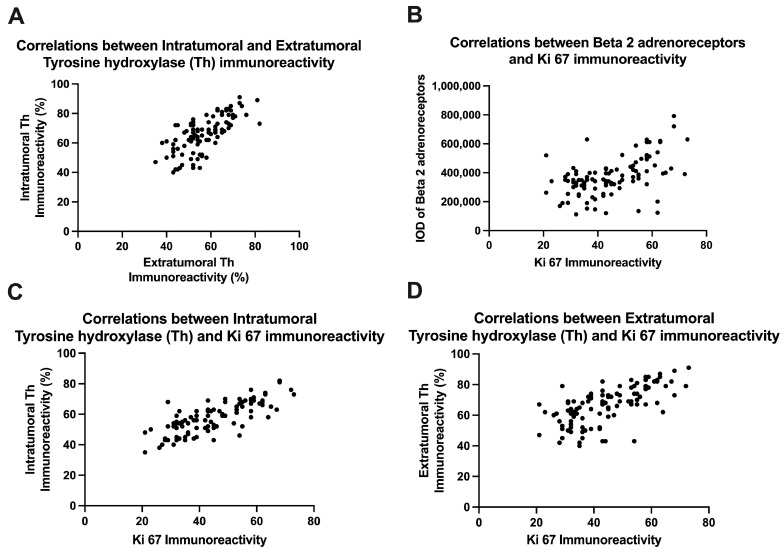
Correlations between intra-tumoral and extra-tumoral tyrosine hydroxylase immunoreactivity (**A**), between beta 2 adrenoreceptors and Ki 67 (**B**), between intra-tumoral tyrosine hydroxylase and Ki 67 (**C**), and between extra-tumoral tyrosine hydroxylase and Ki 67 (**D**).

After analyzing the immunoreactivity of the percentage of intra-tumoral and extra-tumoral sympathetic fibers, we noticed that there was a positive correlation between the two categories (r = 0.7235, 95% confidence interval 0.6169 to 0.804, *p* < 0.0001, Figure 6A). Depending on the immunoreactivity of beta 2 adrenergic receptors, we also observed a positive correlation between the increase in immunoreactivity for these receptors and the tumor proliferation index (r = 0.5159, 95% confidence interval 0.3590 to 0.6445, *p* < 0.0001, Figure 6B), thus suggesting a link between these receptors and the degree of tumor proliferation. Moreover, positive correlations were also recorded between the density of intra-tumoral sympathetic fibers and the tumor proliferation index (r = 0.7674, 95% confidence interval 0.6745 to 0.8364, *p* < 0.0001, Figure 6C), and between extra-tumoral sympathetic fibers and the tumor proliferation index (r = 0.6631, 95% confidence interval 0.5394 to 0.7588, *p* < 0.0001, Figure 6D).

**Figure 7:**
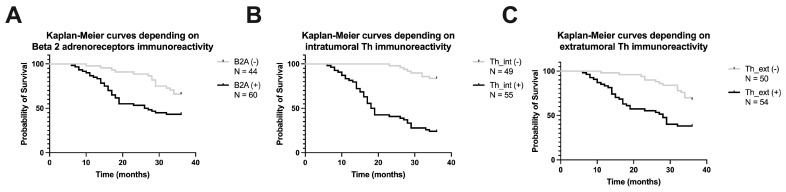
Kaplan–Meier curves depending on beta 2 adrenoreceptors immunoreactivity (**A**), intra-tumoral tyrosine hydroxylase (Th) immunoreactivity (**B**), and extra-tumoral tyrosine hydroxylase (Th) immunoreactivity (**C**).

Regarding the survival analysis of patients with gastric cancer, a better survival rate (65%) was observed for patients with a low value of density of beta 2 adrenergic receptors, categorized as B2A (−) if the value was below the median of the group, compared to B2A (+) patients, in whom the survival rate at 3 years of follow-up was only 43% (Hazard Ratio 2.306, 95% CI of ratio 1.317 to 4.037, *p* = 0.0013, Figure 7A). With regard to the analysis of intra-tumoral sympathetic fibers, a better survival rate (83%) was observed for patients with a low value of sympathetic density, categorized as Th_int (−) if the value was below the median of the group, compared to Th_int (+) patients, in whom the survival rate at 3 years of follow-up was only 24% (Hazard Ratio 8.117, 95% CI of ratio 4.568 to 14.42, *p* < 0.0001, Figure 7B). The same observations were valid for the analysis of extra-tumoral sympathetic fibers, where a better survival rate (68%) was observed for patients with a low value of sympathetic density, categorized as Th_ext (−) if the value was below the median of the group, compared to Th_ext patients (+), in whom the survival rate at 3 years of follow-up was only 38% (Hazard Ratio 2.868, 95% CI of ratio 1.626 to 5.060, *p* < 0.0001, Figure 7C).

## 4. Discussion

Overall, in our study, we found an increased expression of beta 2 adrenoreceptors in poorly differentiated tumors. Although the same observation could not be made about the density of sympathetic nerve fibers depending on the tumor grading, we still found that they were correlated with a lower survival rate in patients who presented a higher density of sympathetic fibers.

A relationship between the autonomic nervous system and stomach cancer certainly exists, and this relationship has attracted the attention of researchers in recent years, but the mechanisms by which the nervous system is involved in gastric tumorigenesis, as well as in tumor progression and metastasis, are still only partially understood. It is certain that in order to understand these mechanisms, the signaling molecules between nerves and tumors must be analyzed (in our case, it was the sympathetic fibers that released norepinephrine), as well as the receptors for these molecules (in our case, the beta 2 adrenergic receptors) expressed by the tumor cells.

Recent studies have shown that the sympathetic nervous system is involved in many types of cancer [21,36,37,38,39]. This subdivision of the nervous system acts through noradrenaline, which is released by the local sympathetic fibers in the vicinity of or even inside the gastric tumors and through epinephrine released from the adrenal gland [21]. In this type of cancer, the most frequently expressed receptors are beta 2 adrenoreceptors [22]. The release of norepinephrine from sympathetic nerve fibers through the action on beta 2 adrenoreceptors activates stimulatory G proteins (Gs) [40]. This activation determines the excess production of cyclic adenosine monophosphate (cAMP) with the activation of protein kinase A (PKA) and the stimulation of tumor cell proliferation, migration, and metastasis [40]. Moreover, in a recent study, Qi et al. showed that in the tumor microenvironment of stomach adenocarcinoma, there is an increased concentration of norepinephrine and a slight increase in beta 3 adrenoreceptors, but predominantly an increase in beta 2 adrenoreceptors and the absence of expression of beta 1 adrenoreceptors [38]. These observations were also confirmed in our study. Moreover, we showed that they correlate negatively with the survival of patients with this type of neoplasia.

Another possible method of interaction between nerves and stomach tumors can be represented by macrophages associated with the tumor microenvironment, polarized in such a way as to allow tumor growth, angiogenesis, and even metastasis [41]. The stimulation of beta 2 adrenoreceptors causes the suppression of macrophages involved in anti-tumor defense and the stimulation of macrophages that favor tumor growth via chitinase-3-like protein 1 (CHI3L1), the transcription factor GATA3, or tumor suppressor protein p 53 (TP53) [41,42,43].

Moreover, there can be a bidirectional influence because the relationship between the nervous system and stomach cancer is complex and multifaceted [39]. Neurons or enteric glial cells can secrete neurotransmitters or related substances that can control the development of stomach tumors [39,44]. Similarly, neural-related substances originating from stomach cancer can impact neural reprogramming, the recruitment of new neural cells, and the growth of new nerve fibers [39]. This process is aided by various neurotrophic factors, axon guidance molecules, and neurotransmitters [39].

Ultimately, a thorough understanding of neuro-tumor regulation offers valuable insights and potential pathways for cancer treatment. The intricate interplay between the nervous system and tumors is widely acknowledged as a pivotal determinant in tumor advancement and treatment efficacy. Exploring neuroregulatory mechanisms can reveal new targets and pathways that can be used as a foundation for creating innovative treatment strategies. An example in this sense is represented by the resistance to trastuzumab that occurs through the upregulation of beta 2 adrenergic receptors in breast cancer [45]. Experimental therapy with beta blockers associated with trastuzumab demonstrated a better survival of breast cancer patients [46].

From a perspective standpoint [47], gaining a profound understanding of the interplay between nerves and stomach tumors can yield novel perspectives and methodologies for the treatment of cancer. This progress will be driven by the advancement of clinical interventions, moving away from the historically challenging denervation surgeries of the 19th century [39] towards contemporary supplementary therapies that specifically target tumor innervation.

### Limitations of This Study

This study has certain limitations. First, the size of the patient cohort was relatively small, which may limit the precision of certain assessments, such as the correlation between specific clinicopathological features and survival. Thus, the ability to generalize the results of our study has been diminished. Second, this study is retrospective, and it only includes data from human patients without comparisons to preclinical studies on cell lines or animal models. Thus, the understanding of the underlying mechanisms of this neuro-neoplastic interaction in stomach cancer is limited.

## 5. Conclusions

The findings of this study indicate that patients with stomach cancer have a more unfavorable prognosis when they have a higher density of sympathetic nerve fibers and an increased expression of beta 2 adrenergic receptors inside the tumor. However, further research on cell lines or animal models and much larger cohorts of patients is needed to validate the results of our study.

## Figures and Tables

**Table 1:** Clinicopathological features.

Clinicopathological Features		n	Beta 2 Adrenoreceptors IOD Mean ± St.dev.	*p*-Value *
Age group	<60	51	395,922 ± 148,315	0.2147
	≥60	53	362,902 ± 121,092	
Gender	Female	41	411,724 ± 136,031	0.1183
	Male	63	368,106 ± 139,271	
Location	Cardia	21	361,584 ± 154,098	0.3909
	Gastric body or pyloric area	83	337,106 ± 105,039	
Histology	Adenocarcinoma	82	368,927 ± 143,073	0.7107
	Mixed carcinoma/signet ring cell carcinoma	22	356,653 ± 113,033	
Tumor size	<5 cm	60	410,932 ± 160,351	0.0371
	≥5 cm	44	481,605 ± 179,328	
Tumor invasion	T_1–2_	39	367,060 ± 156,373	0.0159
	T_3–4_	65	453,943 ± 185,764	
Lymph node metastasis	N_0–1_	43	399,547 ± 140,174	0.0462
	N_≥2_	61	459,933 ± 156,909	
TNM stage	T_I–II_	42	357,243 ± 117,430	0.0119
	T_III–IV_	62	437,908 ± 179,690	

* *t*-test.

## Data Availability

Data are contained within this article or available upon request from the corresponding authors.
